# Comparative organellar genomics of *Arundina graminifolia*: mitochondrial complexity and plastid conservation in Orchidaceae

**DOI:** 10.3389/fpls.2026.1756243

**Published:** 2026-02-12

**Authors:** Shengnan Lin, Chao Song, Jie Wang, Jiali Kong, Dan Peng, Jie Gao, Zhiqiang Wu

**Affiliations:** 1 Kunpeng Institute of Modern Agriculture at Foshan, Chinese Academy of Agricultural Sciences, Shenzhen, China; 2 Guangdong Laboratory of Lingnan Modern Agriculture, Agricultural Genomics Institute at Shenzhen, Chinese Academy of Agricultural Sciences, Shenzhen, China; 3 State Key Laboratory of Tropical Crop Breeding, Ministry of Agriculture and Rural Affairs, Agricultural Genomics Institute at Shenzhen, Chinese Academy of Agricultural Sciences, Shenzhen, China; 4 Guangdong Laboratory of Lingnan Modern Agriculture, Ministry of Agriculture and Rural Affairs, Agricultural Genomics Institute at Shenzhen, Chinese Academy of Agricultural Sciences, Shenzhen, China; 5 Key Laboratory of Synthetic Biology, Ministry of Agriculture and Rural Affairs, Agricultural Genomics Institute at Shenzhen, Chinese Academy of Agricultural Sciences, Shenzhen, China; 6 Chongqing Key Laboratory for Germplasm Innovation of Special Aromatic Spice Plants, College of Smart Agriculture, Chongqing University of Arts and Sciences, Chongqing, China; 7 School of Ecology, Hainan University, Haikou, China; 8 Guangdong Key Laboratory of Ornamental Plant Germplasm Innovation and Utilization, Environmental Horticulture Research Institute, Guangdong Academy of Agricultural Sciences, Guangzhou, China

**Keywords:** genome evolution, mitochondrial genome, Orchidaceae, organelle genomes, plastid genome

## Abstract

Orchidaceae is one of the largest and most diverse angiosperm families, exhibiting remarkable morphological and ecological diversity. Organellar genomes, including mitochondrial and plastid genomes, play essential roles in energy metabolism, photosynthesis, and adaptive evolution, yet their structural evolution in orchids remains unclear. In this study, the mitochondrial and plastid genomes of *Arundina graminifolia* were assembled and compared with 15 representative orchid species to investigate genome architecture, repeat content, gene composition, and evolutionary dynamics. The mitochondrial genome of *A. graminifolia* displayed a complex multi-circular structure with extensive rearrangements and abundant repeats, including simple sequence repeats and long repeats, contributing to genome expansion and structural complexity. In contrast, the plastid genome was highly conserved, exhibiting a typical quadripartite structure. Plastid-to-mitochondrion DNA transfers (MTPTs) varied among orchid species in both number and composition, mainly involving complete plastid genes associated with photosynthesis and translation. Analyses of selection pressure and nucleotide diversity revealed strong purifying selection and low sequence variability in mitochondrial genes, whereas plastid genes exhibited higher diversity and evolutionary rates. Phylogenetic analyses based on conserved single-copy genes from both organellar genomes yielded congruent topologies, with *A. graminifolia* clustering closely with *Bletilla striata*. Synteny analysis indicated low conservation of mitochondrial genome organization across lineages, particularly in mycoheterotrophic orchids, reflecting dynamic genome evolution. Gene loss and duplication further enhanced genome plasticity and potential functional redundancy. Overall, this study highlights contrasting evolutionary constraints and structural dynamics between mitochondrial and plastid genomes in orchids, providing valuable genomic resources for understanding organelle genome evolution and phylogenetic relationships in Orchidaceae.

## Introduction

The Orchidaceae is one of the largest and most diverse families of angiosperms, encompassing over 28,000 species that are widely distributed across tropical, subtropical, and temperate regions (Chase et al., 2015; Christenhusz, 2016; Zhang et al., 2022). Orchid species exhibit remarkable morphological and ecological diversity, including complex reproductive strategies, diverse life forms (terrestrial, epiphytic, or mycoheterotrophic), and highly specialized floral structures, making them an important model for studying plant evolution, adaptation, and ecological function (Dressler, 1981; Dressler, 1993; Rasmussen and Rasmussen, 2009; Givnish et al., 2015; Zhang et al., 2018). Therefore, investigating their genome features not only enhances understanding of Orchidaceae evolution but also provides molecular foundations for conservation, breeding, and sustainable utilization.

Organellar genomes, including mitochondrial and plastid genomes, play central roles in plant energy metabolism, photosynthesis, and essential biochemical pathways (Gray et al., 1999; Neuhaus and Emes, 2000; Woodson and Chory, 2008). Plastid genomes are typically highly conserved, exhibiting a quadripartite structure composed of large single-copy (LSC) and small single-copy (SSC) regions separated by two inverted repeats (IRs), typically with stable gene content and limited gene loss or duplication events (Raubeson and Jansen, 2005; Wicke et al., 2011; Jansen and Ruhlman, 2012; Wang et al., 2024b). In contrast, plant mitochondrial genomes are highly dynamic, characterized by complex structures, frequent gene rearrangements, abundant repeat sequences, and incorporation of foreign DNA fragments (Wang et al., 2007; Park et al., 2015; Sun et al., 2022; Wu et al., 2022; Wang et al., 2024c). These features result in substantial interspecific variation in size, structure, and gene content of mitochondrial genome, shaped by complex selective pressures over the course of evolution.

Recent advances in high-throughput sequencing technologies have facilitated the assembly and annotation of numerous orchid organelle genomes, providing valuable data for comparative genomics and phylogenetic studies (Park et al., 2015; Kim et al., 2020; Li et al., 2024a; Shen et al., 2024; Wu et al., 2024; Soe et al., 2025). However, most studies have focused either on plastid genomes or individual mitochondrial genomes, and few have systematically analyzed the evolutionary dynamics of both organelle genomes simultaneously. In particular, the roles of repeat expansion, plastid-to-mitochondrion DNA transfer (MTPTs), gene loss, and gene duplication in shaping genome size, structural diversity, and genetic variation across species remain largely unexplored (Cole et al., 2018; Yang et al., 2023; Wang et al., 2024a). A comprehensive investigation of these aspects is essential for understanding the evolution, functional maintenance, and ecological adaptation of organelle genomes in Orchidaceae.

*Arundina graminifolia*, a tropical orchid with high ornamental value (Ahmad et al., 2021; Li et al., 2025a), lacks complete organelle genome information. To address this gap, the present study assembled and annotated the mitochondrial and plastid genomes of *A. graminifolia* and conducted comparative analyses with 15 representative orchid species. Specifically, the study aimed to: (1) characterize the overall genome structure, repeat sequences, and plastid DNA transfer events to assess their contributions to genome expansion and structural complexity; (2) evaluate selection pressures (Ka/Ks) and nucleotide diversity (Pi) of conserved single-copy genes in mitochondrial and plastid genomes to reveal evolutionary patterns and functional constraints; and (3) reconstruct phylogenetic relationships using single-copy genes and assess genome synteny to explore structural evolution. Collectively, these analyses provide a genomic framework for understanding organelle genome evolution, functional adaptation, and phylogenetic relationships in Orchidaceae, offering valuable insights for further comparative genomics, molecular breeding, and ecological adaptation studies.

## Materials and methods

### Data source

The *A. graminifolia* sample used for sequencing was obtained from the greenhouse of the Environmental Horticulture Research Institute, Guangdong Academy of Agricultural Sciences. Whole-genome HiFi sequencing was performed by Kindstar Sequenon Biotechnology (Wuhan) Co., Ltd, and the mitochondrial and plastid genomes were subsequently assembled by our team from the resulting unpublished data. Organelle genome sequences of 15 additional Orchidaceae species were downloaded from national center for biotechnology information (NCBI) (**Supplementary Table S1**). These sequences were used for subsequent analyses, including repeat identification, MTPTs, Ka/Ks and Pi analyses, phylogeny reconstruction, and synteny analyses.

### *A. graminifolia* organelle genome assembly and annotation

The mitochondrial genome of *A. graminifolia* was assembled using two complementary approaches. The full set of whole-genome HiFi sequencing reads was processed using HiMT 1.1.1 (Tang et al., 2025) for organelle genome assembly from long-read data. A 30% random subset of the HiFi reads (~19 Gb) was subjected to assembly using Tippo 1.3.0 (Xian et al., 2025) for efficient reconstruction of organelle genomes with high fidelity. Candidate contigs were filtered with CD-HIT 4.8.1 (Li and Godzik, 2006; Fu et al., 2012) with default parameters and then validated against conserved mitochondrial genes and related genomes using BLAST 2.15.0+ (Madden, 2013) with an E-value cutoff of 1×10^−5^. Contigs supported by both methods were merged and polished with Pilon 1.24 (Walker et al., 2014). Plastid assemblies were generated concurrently, with consistency verified through collinearity analysis.

A combined strategy of automated prediction using PMGA (http://www.1kmpg.cn/pmga/) (Li et al., 2025b) and manual curation in Apollo 1.11.8 (Lewis et al., 2002) was adopted for mitochondrial genome annotation with published mitochondrial genes and rRNAs as references. tRNAs were predicted using tRNAscan-SE 2.0 (Chan et al., 2021) with default parameters. plastid annotation was performed with CPGAVAS2 (http://47.96.249.172:16019/analyzer/home) (Shi et al., 2019) and manually verified in Apollo 1.11.8 (Lewis et al., 2002). Genome maps were visualized using ShinyCircos 2.0 (https://venyao.xyz/shinycircos/) (Wang et al., 2023) for the mitochondrial genome and OGDRAW 1.3.1 (https://chlorobox.mpimp-golm.mpg.de/OGDraw.html) (Greiner et al., 2019) for the plastid genome.

### Codon usage and nucleotide composition analysis

The sequences of protein-coding genes (PCGs) were extracted from the mitochondrial genome using PhyloSuite 2.0 (Zhang et al., 2020). Codon usage and nucleotide composition analyses were performed with CodonW 1.4.4 (Sharp and Li, 1986) under default settings to calculate GC content, relative synonymous codon usage (RSCU), and the effective number of codons (ENC). ENC-GC3 plots were generated using the R package ggplot2 (Wickham, 2011), with ENC values on the y-axis and GC content at the third codon position (GC3) on the x-axis. The expected ENC curve was calculated according to previous research (Romero et al., 2000), using the formula:


$$ENC\;=\;2\;+\;GC3\;+\;\frac{29}{\left(GC3^{2}+\;{\left(1\;-\;GC3\right)}^{2}\right)}$$

Genes located on or near the expected curve indicate that codon usage bias is mainly shaped by mutational pressure, whereas genes below the curve suggest that natural selection plays a dominant role (Sueoka, 1988). Additionally, the scatter plots of GC content were generated using ggplot2 (Wickham, 2011), with the mean GC content at the first and second codon positions (GC12) on the y-axis and GC3 on the x-axis.

### Repeat sequence and MTPTs identification

Repeat sequences in *A. graminifolia* and other 15 Orchidaceae mitochondrial genomes were identified by self-alignment using BLAST 2.15.0+ (word size = 7, E-value ≤ 1 × 10^−5^) (Madden, 2013). The repeat sequences with length ≥ 30 bp and sequence similarity ≥ 80% were retained, and classified into four retained and classified into five categories: Class I (30 ≤ L ≤ 100 bp), Class II (100 < L ≤ 200 bp), Class III (200 < L ≤ 500 bp), Class IV (500 < L ≤ 1000 bp), and Class V (> 1000 bp). Simple sequence repeats (SSRs) were detected using MISA (https://webblast.ipk-gatersleben.de/misa/index.php?action=1) (Beier et al., 2017), with minimum repeat thresholds of 10, 8, 5, 4, 3, and 3 for mono-, di-, tri-, tetra-, penta-, and hexanucleotide motifs, respectively. For each species, the total number and cumulative length of repeats were calculated, excluding shorter repeats nested within longer ones. Spearman correlations between repeat abundance, cumulative repeat length, and mitochondrial genome size were analyzed and visualized using ggplot2 (Wickham, 2011).

MTPTs were identified by aligning the high-quality genome of plastid of each species against its corresponding mitochondrial assemblies using BLASTN 2.15.0+ (Madden, 2013) with the threshold: E-value ≤ 1×10^−5^, alignment length ≥ 250 bp, sequence identity ≥ 80%, and word size = 7. Candidate MTPTs were filtered to remove redundancy by merging overlapping or adjacent fragments. Each retained MTPT was annotated to determine genomic position, length, sequence similarity, and whether it contained full-length or partial plastid coding genes.

### Evolutionary and comparative analyses

The rates of nonsynonymous (Ka) and synonymous (Ks) substitutions in mitochondrial genomes were estimated using single-copy orthologs from *A. graminifolia* and other 15 Orchidaceae species. Homologous sequences were aligned with ParaAT 2.0 (Zhang et al., 2012) with default parameters., and Ka/Ks values were calculated using the ‘Simple Ka/Ks Calculator’ in TBtools-II (Chen et al., 2023). Nucleotide diversity (Pi) in plastid genomes was assessed from single-copy orthologous genes aligned with MAFFT 7.429 (Katoh and Standley, 2013) under default settings, and Pi values were calculated using DnaSP 6.0 (Rozas et al., 2017) with a sliding window of 100 bp and step size of 50 bp. Results were visualized in R using ggplot2 (Wickham, 2011).

Phylogenetic relationships were inferred using 11 mitochondrial and 8 plastid single-copy genes with *Allium cepa* as the outgroup. Nucleotide sequences were translated to amino acids, aligned with MAFFT 7.429 (Katoh and Standley, 2013), and back-translated using pal2nal.pl (Suyama et al., 2006). Low-quality regions were trimmed with TrimAl (Capella-Gutiérrez et al., 2009), and sequences were concatenated into a supermatrix with Python script. Maximum likelihood phylogenies were generated using IQ-TREE 3.0.1 (Wong et al., 2025), with ultrafast bootstrap method to assess nodal support. Amino acid-based phylogenies were generated following the same workflow above.

Synteny among organelle genomes was analyzed using 16 mitochondrial genomes. Whole-genome alignments were performed with nucmer (--maxmatch), filtered with delta-filter for one-to-one matches, and coordinates were extracted with show-coords (Marçais et al., 2018). Genome synteny link files were generated using GetTwoGenomeSyn.pl and visualized in NGenomeSyn 1.43 (He et al., 2023), enabling systematic comparison of structural conservation and rearrangements across Orchidaceae mitochondrial genomes.

## Results

### Organellar genome features of *A. graminifolia*

**Figure 1:**
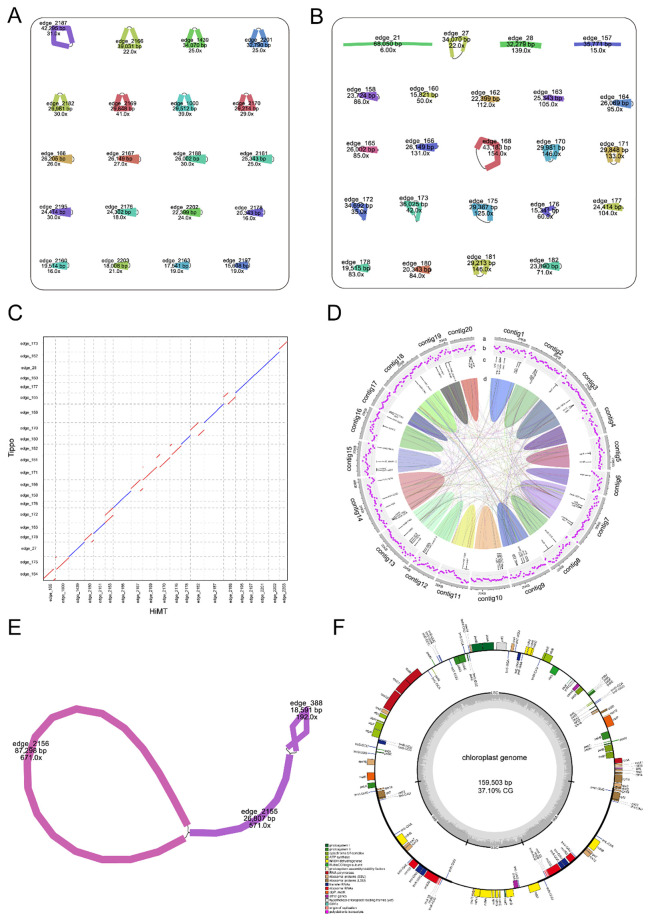
Assembly and structural features of the *A. graminifolia* mitochondrial and plastid genomes. **(A)** Structural overview of the mitogenome assembled using the HiMT software. **(B)** Structural overview of the mitogenome assembled using the Tippo software. **(C)** Synteny comparison between the HiMT- and Tippo-assembled mitogenomes. Forward alignments are shown in red, and reverse alignments are shown in blue. **(D)** Circular annotation map of the mitogenome. Panels: **(a)** twenty contigs of the mitochondrial genome; **(b)** GC content distribution; **(c)** annotated protein-coding genes, rRNAs, and tRNAs; **(d)** internal repeat-based synteny. **(E)** Structural overview of the plastid genome of *A*. *graminifolia*. **(F)** Circular annotation map of the *A*. *graminifolia* plastid genome.

To characterize the organelle genomes of *A. graminifolia*, we assembled and annotated its mitochondrial and plastid genomes using two independent *de novo* assemblers, HiMT and Tippo. The mitochondrial genome assembly with HiMT produced 20 circular molecules, whereas the Tippo assembly yielded 20 circular molecules and 3 linear fragments (**Figures 1A, B**). Synteny analysis revealed that edge_2166 in HiMT corresponded to the concatenation of edge_176 and edge_158 in Tippo, while edge_21 and edge_157 from Tippo lacked corresponding sequences in HiMT (**Figure 1C**, **Supplementary Table S2**). The fragments edge_21 and edge_157, which contained no identifiable coding genes, showed markedly lower sequencing support compared with other mitochondrial contigs, and were thus excluded from downstream analyses. Overall, mitochondrial assemblies were highly consistent when using different methods, and the plastid genome assemblies were identical. Consequently, the HiMT assembly was adopted as the reference for subsequent analyses.

The mitochondrial genome displayed a multicircular structure with a total length of 532,569 bp and a GC content of 44.0% (**Figure 1D**, **Supplementary Table S3**). It encoded 27 core protein-coding genes, 13 variable protein-coding genes, 22 tRNA genes, and 3 rRNA genes, including ATP synthase (*atp1*–*atp9*), cytochrome *c* biogenesis (*ccmB*, *ccmC*, *ccmFC*, *ccmFN*), cytochrome *b* (*cob*), cytochrome c oxidase (*cox1*–*cox3*), NADH dehydrogenase (*nad1*–*nad9*), succinate dehydrogenase (*sdh4*), maturase (*matR*), and membrane transport protein (*mttB*) genes (**Table 1**). Several genes contained 1–4 introns, including *ccmFC*, *rps3*, *rps10*, *cox2*, *nad1*, *nad2*, *nad4*, *nad5*, and *nad7*.

**Table 1:** Gene composition of the *A. graminifolia* mitochondrial genome.

Gene category	Functional group	Name of genes
Core genes	ATP synthase	*atp1* (2), *atp4*, *atp6*, *atp8*, *atp9* (2)
	Cytochrome c biogenesis	*ccmB*, *ccmC*, ccmFC*, *ccmFN*
	Ubiquinol cytochrome c reductase	*cob*
	Cytochrome c oxidase	*cox1*, cox2**, *cox3*
	Maturases	*matR*
	Transport membrane protein	*mttB*
	NADH dehydrogenase	nad1****, nad2****, *nad3*, nad4***, nad4L, nad5*, *nad6*, nad7***, *nad9*
	Subunit of succinate dehydrogenase	*sdh4*
Variable genes	Ribosomal proteins (LSU)	*rpl2*, *rpl5*, *rpl16*
	Ribosomal proteins (SSU)	*rps1*, *rps2*, rps3*, *rps4*, *rps7*, rps10*, *rps12*, *rps13*, *rps14*, *rps19*
rRNA	Ribosomal RNAs	*rrn18*, *rrn26*, *rrn5*
tRNA	Transfer RNAs	trnE-UUC* (2), trnM-CAU* (3), trnT-GGU*, *trnC-GCA* (2), *trnD-GUC*, *trnF-GAA* (2), *trnH-GUG* (2), *trnK-UUU*, *trnL-UAG*, *trnI-CAU*, *trnfM-CAU*, *trnN-GUU*, *trnQ-UUG*, *trnW-CCA*, *trnY-GUA*, trnA-UGC*

Asterisks (*) indicate the number of introns, and numbers in parentheses indicate the copy number of multi-copy genes.

The plastid genome exhibited the typical quadripartite structure, consisting of LSC, SSC, and a pair of IRs, with a total length of 159,503 bp and a GC content of 37.10% (**Figure 1E**). The genome contained 81 protein-coding genes, 27 tRNA genes, and 8 rRNA genes. Several of these genes are duplicated in the IR regions, including *ndhB*, *rpl2*, *rpl23*, *rps7*, *rps12*, and *rps19*. Nine genes contained a single intron, and three genes contained two introns (**Figure 1F**, **Table 2**). These high-quality assemblies provide a foundation for comparative and evolutionary analyses in Orchidaceae.

**Table 2:** Gene composition of the *A. graminifolia* plastid genome.

Gene category	Functional group	Name of genes
Photosynthesis	Subunits of ATP synthase	*atpA*, *atpB*, *atpE*, atpF*, *atpH*, *atpI*
	Subunits of photosystem I	*psaA*, *psaB*, *psaC*, *psaI*, *psaJ*
	Subunits of photosystem II	*psbA*, *psbB*, *psbC*, *psbD*, *psbE*, *psbF*, *psbH*, *psbI*, *psbJ*, *psbK*, *psbL*, *psbM*, *psbN*, *psbT*, *psbZ*
	Subunits of NADH-dehydrogenase	ndhA*, ndhB* (2), *ndhC*, *ndhD*, *ndhE*, *ndhF*, *ndhG*, *ndhH*, *ndhI*, *ndhJ*, *ndhK*
	Subunits of cytochrome b/f complex	*petA*, petB*, petD*, *petG*, *petL*, *petN*
	Subunits of rubisco	*rbcL*
Self-replication	Large subunit of ribosome	rpl2* (2), *rpl14*, rpl16*, *rpl20*, *rpl22*, *rpl23* (2), *rpl33*, *rpl36*
	DNA dependent RNA polymerase	*rpoA*, *rpoB*, rpoC1*, *rpoC2*
	Small subunit of ribosome	*rps2*, *rps3*, *rps4*, *rps7* (2), *rps8*, *rps11*, rps12** (2), *rps14*, *rps15*, rps16**, *rps18*, *rps19* (2)
	Ribosomal RNAs	*rrn4.5S* (2), *rrn5S* (2), *rrn16S* (2), *rrn23S* (2)
	Transfer RNAs	trnA-UGC* (2), *trnC-GCA*, *trnD-GUC*, trnE-UUC* (3), *trnF-GAA*, *trnG-GCC*, *trnH-GUG* (2), *trnL-CAA* (2), trnL-UAA*, *trnL-UAG*, *trnM-CAU* (4), *trnN-GUU* (2), *trnP-UGG*, *trnQ-UUG*, *trnR-ACG* (2), *trnR-UCU*, trnS-CGA*, *trnS-GCU*, *trnS-GGA*, *trnS-UGA*, *trnT-GGU*, *trnT-UGU*, *trnV-GAC* (2), *trnW-CCA*, *trnY-GUA*
	c-type cytocgrom synthesis gene	*ccsA*
	Envelop membrane protein	*cemA*
	Translational initiation factor	*infA*
	Protease	clpP**
	Maturase	*matK*
Unkown	Conserved open reading frames	ycf3**, *ycf4*

Asterisks (*) indicate the number of introns, and numbers in parentheses indicate the copy number of multi-copy genes.

### Codon usage in *A. graminifolia* mitogenome

**Figure 2:**
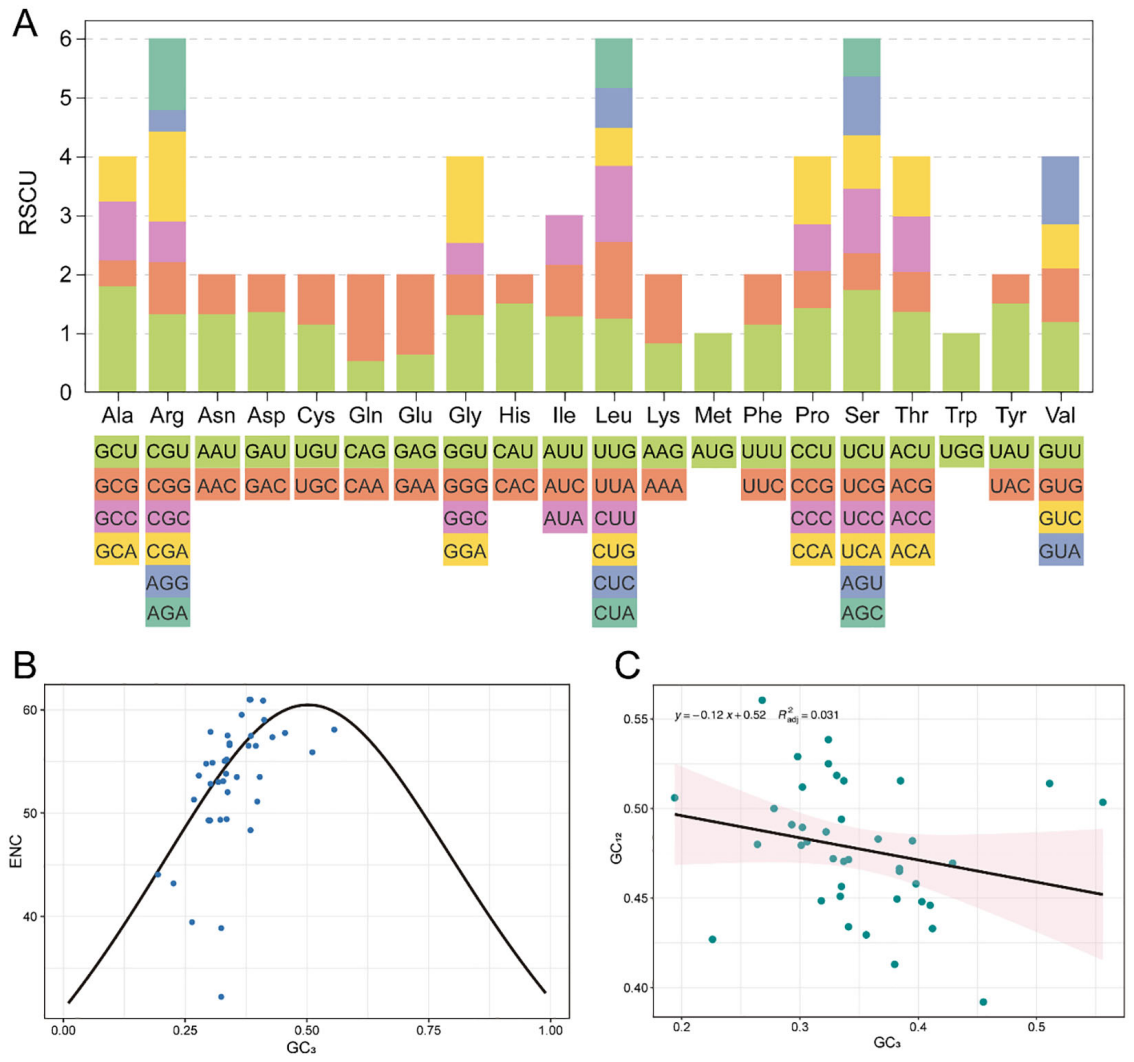
Codon usage and selection pressure analysis of protein-coding genes in the mitochondrial genome of *A*. *graminifolia*. **(A)** RSCU analysis. **(B)** ENC scatter plot of protein-coding genes. **(C)** Neutrality plot analysis of protein-coding genes.

Codon usage analysis of the 40 mitochondrial protein-coding genes yielded 10,213 codons. Leucine (Leu) was the most abundant amino acid (10.58%), followed by serine (Ser, 8.38%), while the stop codons were the least frequent (0.13%) (**Supplementary Table S4**). RSCU analysis indicated that the most preferred codons ended with A/U, with strong preferences for Ala (GCU, RSCU = 1.97), Ser (UCU, RSCU = 1.73), and Arg (GCU, RSCU = 1.53) (**Figure 2A**, **Supplementary Table S4**).

GC content analysis showed uneven distribution across codon positions: GC1 (21.57%), GC2 (22.13%), GC3 (34.86%), and overall GC (43.47%) (**Supplementary Table S5**). ENC values ranged from 32.22 to 61, with an average of 46.92, and the two *atp9* copies displayed the lowest (32.22) and second lowest ENC values, reflecting strong codon usage bias. ENC scatterplot analysis suggested that natural selection, rather than mutational pressure, primarily shapes codon usage (**Figure 2B**), further supported by a weak correlation between GC3 and GC12 (slope = 0.12, R² =0.031, **Figure 2C**).

### Gene loss and duplications in Orchidaceae organelle genomes

**Figure 3:**
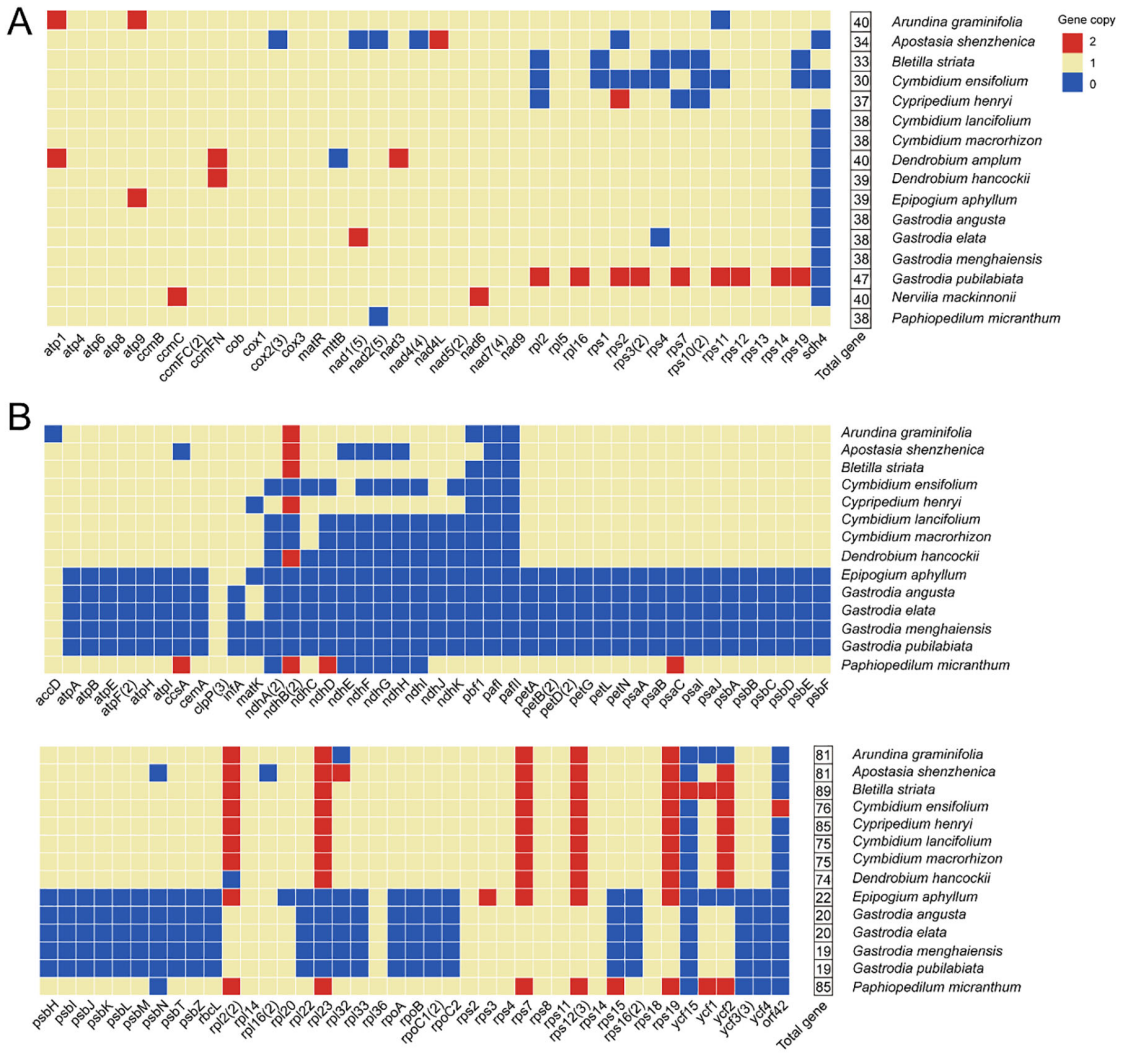
Distribution of protein-coding genes in Orchidaceae organelle genomes. Distribution of protein-coding genes in Orchidaceae mitochondrial **(A)** and plastid **(B)** genomes; numbers in parentheses indicate exon counts.

To explore organellar gene evolution in Orchidaceae, we examined mitochondrial genomes from 16 species together with plastid genomes from 14 species (plastomes were not available for two taxa), and assessed patterns of gene loss and multi-copy gene distribution (**Supplementary Table S1**). In the mitochondrial genomes, most protein-coding genes were highly conserved, particularly those associated with energy metabolism and electron transport, including ATP synthase genes (*atp1*, *atp4*, *atp6*, *atp8*, and *atp9*), cytochrome c biogenesis genes (*ccmB*, *ccmC*, *ccmFC*, and *ccmFN*), the maturase gene (*matR*), and cytochrome b (*cob*) (**Figure 3A**). In contrast, ribosomal protein genes and succinate dehydrogenase subunit genes displayed greater variability, with *rpl2*, *rps1*, *rps2*, *rps3*, *rps4*, *rps7*, *rps10*, *rps11*, and *sdh4* absent in some species. Notably, *rps11* was missing in *A. graminifolia*, *cox2* was lost only in *Apostasia shenzhenica*, and *mttB* was absent exclusively in *Dendrobium amplum*. Among the analyzed species, *Cymbidium ensifolium* harbored the fewest mitochondrial protein-coding genes (30), whereas *Gastrodia pubilabiata* contained the most (47). Several species, including *Bletilla striata*, *Cymbidium* spp., *G. angusta, G. menghaiensis*, and *Paphiopedilum micranthum*, maintained only single-copy mitochondrial genes, while other species exhibited 1–9 genes present in two copies. In *A. graminifolia*, both *atp1* and *atp9* were duplicated, with the duplicated copies exhibiting sequence divergence (**Figure 3A**).

Plastid genomes showed greater variability than mitogenomes across selected orchid species, with total gene numbers ranging from 19 to 89 (**Figure 3B**). *B. striata* contained the highest number of plastid genes, including nine duplicated genes (*ndhB*, *rpl2*, *rpl23*, *rps7*, *rps12*, *rps19*, *ycf1*, *ycf2*, and *ycf15*), whereas *Epipogium aphyllum* and *Gastrodia* species had the fewest genes. Across all 14 analyzed species, *clpP*, *rpl14*, *rpl36*, *rps2*, *rps4*, *rps8*, *rps11*, *rps14*, and *rps18* were consistently retained, indicating their high conservation. In contrast, lineage-specific losses and gains were observed for some genes. For instance, *accD* was absent only in *A. graminifolia*, *rpl16* was missing exclusively in *Apostasia shenzhenica*, *ycf15* was detected only in *B. striata*, and *pafI* and *pafII* were found solely in *P. micranthum* (**Figure 3B**). These patterns indicate that energy-related genes are highly conserved, frequent gene losses and copy number variations reflect dynamic organelle genome evolution.

### Repetitive sequence analysis of Orchidaceae mitogenomes

**Figure 4:**
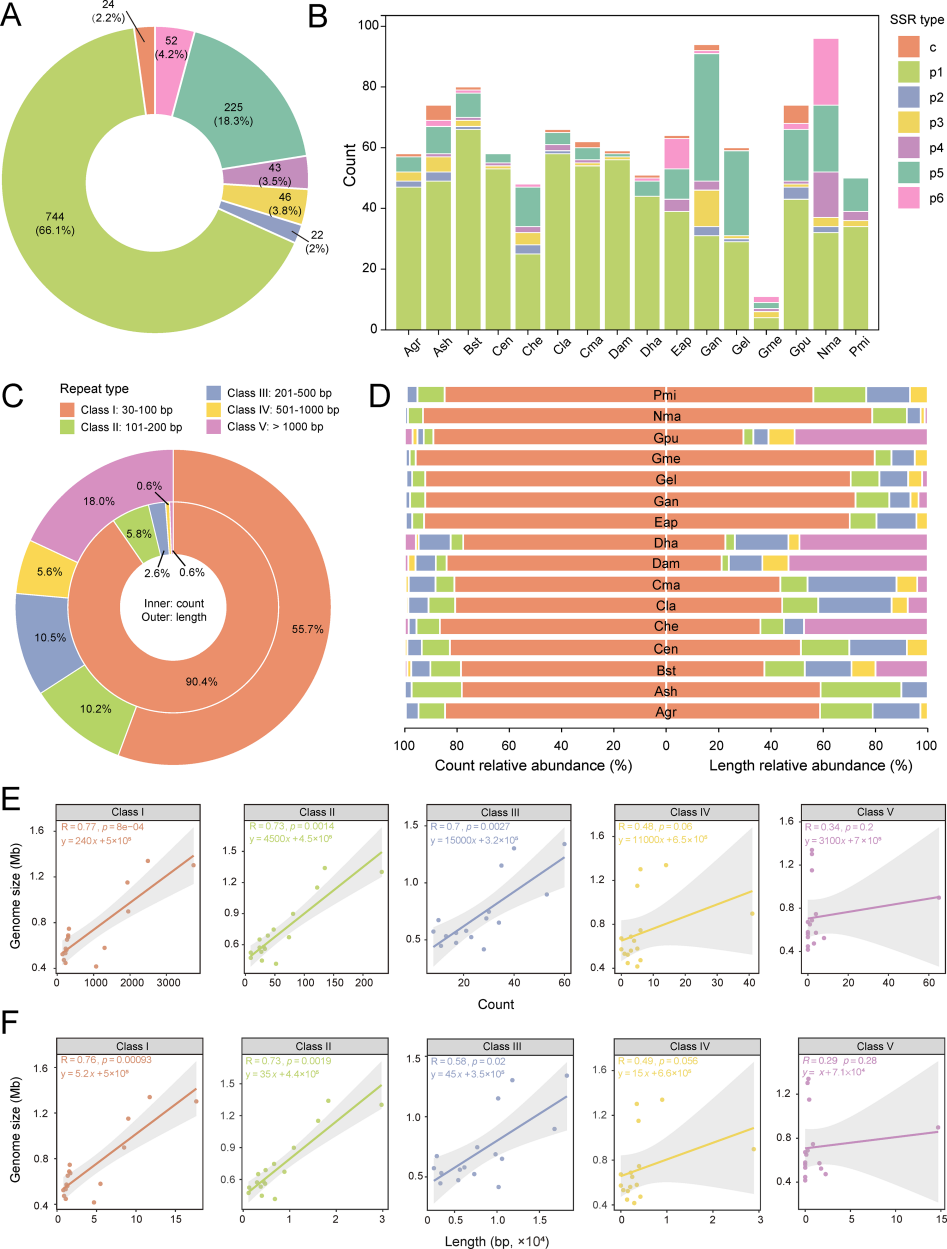
Repeat sequence analysis in mitochondrial genomes of Orchidaceae. **(A)** Overview of SSR types in Orchidaceae mitogenomes. **(B)** SSR types and numbers in each Orchidaceae mitogenome; p1–p6 denote mono-, di-, tri-, tetra-, penta-, and hexanucleotide repeats, respectively, while c represents compound SSRs. **(C)** Overview of repeat sequence types in Orchidaceae mitogenomes. **(D)** Repeat sequence types and numbers in each Orchidaceae mitogenome. **(E, F)** Spearman correlations between repeat abundance or total repeat length and mitogenome size. Species abbreviations used in panels **(B, D)** Agr (*Arundina graminifolia*), Ash (*Apostasia shenzhenica*), Bst (*Bletilla striata*), Cen (*Cymbidium ensifolium*), Che (*Cypripedium henryi*), Cla (*Cymbidium lancifolium*), Cma (*Cymbidium macrorhizon*), Dam (*Dendrobium amplum*), Dha (*Dendrobium hancockii*), Epa (*Epipogium aphyllum*), Gan (*Gastrodia angusta*), Gel (*Gastrodia elata*), Gme (*Gastrodia menghaiensis*), Gpu (*Gastrodia pubilabiata*), Nma (*Nervilia mackinnonii*), Pmi (*Paphiopedilum micranthum*).

A total of 1,156 SSRs were identified from 16 orchid mitogenomes (**Supplementary Table S6**), with mononucleotide repeats being the most abundant (66.1%), followed by pentanucleotide repeats (18.3%), and compound SSRs (2.2%) (**Figure 4A**). Among those Orchid species, *Nervilia mackinnonii* possessed the highest number of SSRs, followed by *G. angusta* (**Figure 4B**, **Supplementary Table S6**). Most species contained seven SSR types, such as *Apostasia shenzhenica*, *B. striata*, *G. angusta*, and *G. pubilabiata*, whereas *C. ensifolium*, *D. amplum*, *D. hancockii*, and *P. micranthum* had only four types (**Figure 4B**, **Supplementary Table S6**). In *A. graminifolia*, 47 mononucleotide, 2 dinucleotide, 3 trinucleotide, 5 pentanucleotide, and 1 compound SSR were detected.

Mitochondrial repeats were further classified by length into five categories: Class I (30–100 bp), Class II (100–200 bp), Class III (200–500 bp), Class IV (500–1000 bp), and Class V (> 1000 bp). Across the 16 mitochondrial genomes (416,775–1,339,825 bp), 16,539 repeats were identified with a cumulative length of 1,252,601 bp (**Supplementary Tables S7**, **S8**). Class I repeats were the most numerous (90.4%) and accounted for the largest proportion of repeat length (55.7%), followed by Class V (18.0%) (**Figure 4C**). Although Class I repeats dominated in most species, Class V contributed disproportionately to total repeat length in *D. hancockii*, *D. amplum*, *Cypripedium henryi*, and *G. pubilabiata* (**Figure 4D**). Some repeat classes were absent in specific species: Class IV repeats were missing in *Apostasia shenzhenica* and *Cypripedium henryi*, and Class V repeats were absent in *A. graminifolia*, *Apostasia shenzhenica*, *C. ensifolium*, *E. aphyllum*, *G. menghaiensis*, and *P. micranthum*. Notably, the mitochondrial genome of *G. pubilabiata* (898,047 bp) contained 65 Class V repeats, far exceeding all other species (< 10), highlighting species-specific expansion of long repeats.

Correlation analysis revealed that mitochondrial genome size in Orchidaceae was strongly positively correlated with the number of Class I–III repeats (R > 0.7, p < 0.001), and also with the total lengths of Class I and II repeats (R > 0.7, p < 0.001). Genome size was additionally positively correlated with Class III repeat length (R > 0.5, p < 0.05) (**Figures 4E, F**). These results highlight the role of repeats in the expansion of the mitochondrial genome and its structural complexity.

### Plastid-derived DNA fragments in Orchidaceaes mitogenomes

**Figure 5:**
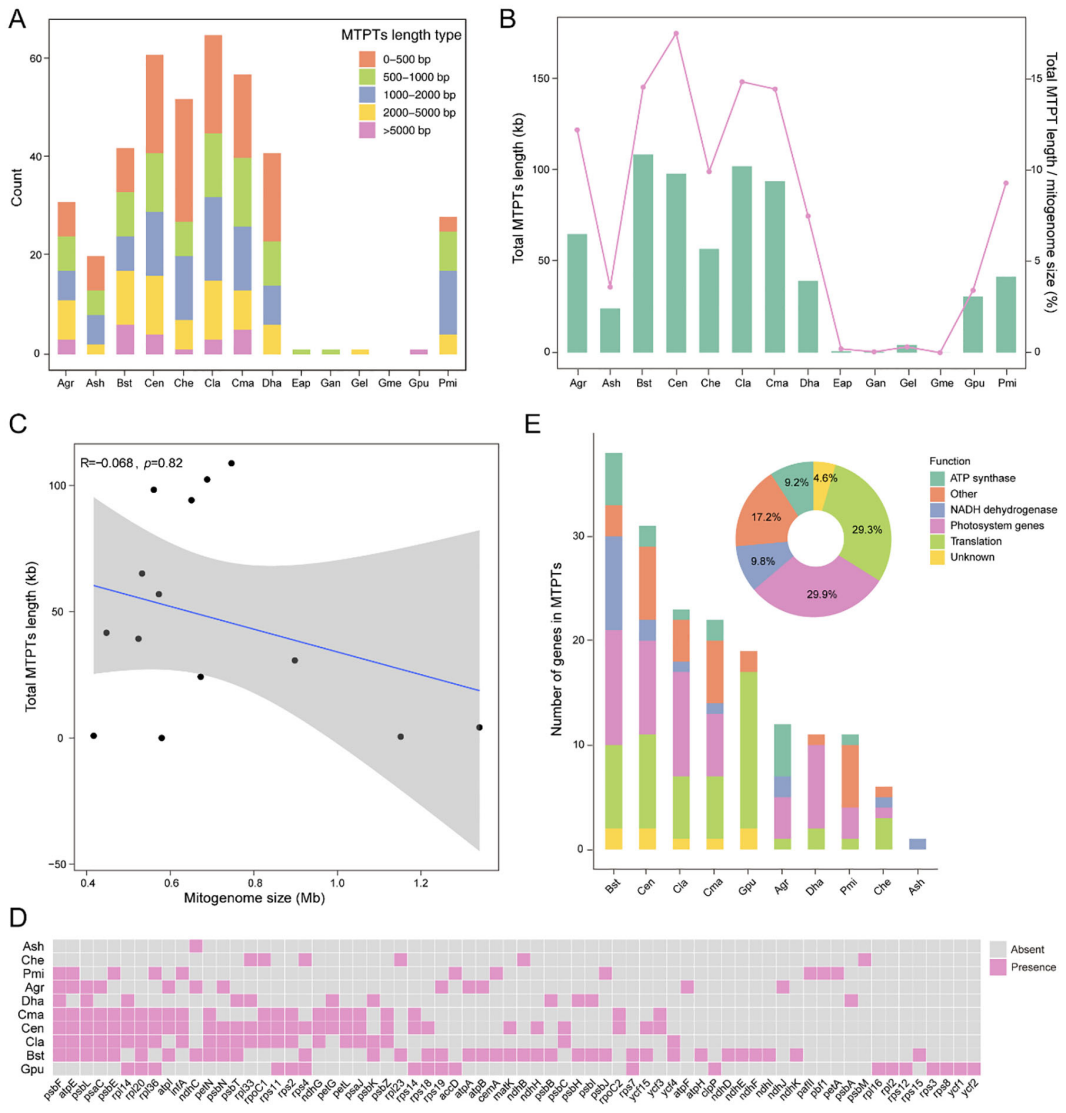
Analysis of MTPTs in Orchidaceae mitochondrial genomes. **(A)** Number of MTPTs of different lengths in Orchidaceae mitochondrial genomes. **(B)** Total length of MTPTs (left) and their ratio to mitochondrial genome size (right). **(C)** Spearman correlation between total MTPT length and mitochondrial genome size. **(D)** Distribution of plastid protein-coding genes within MTPTs. **(E)** Functional classification of plastid protein-coding genes within MTPTs. Pie charts show the overall functional composition, and bar charts display the functional distribution for each mitochondrial genome. Species abbreviations used in panels **(A, B, D)** Agr (*Arundina graminifolia*), Ash (*Apostasia shenzhenica*), Bst (*Bletilla striata*), Cen (*Cymbidium ensifolium*), Che (*Cypripedium henryi*), Cla (*Cymbidium lancifolium*), Cma (*Cymbidium macrorhizon*), Dha (*Dendrobium hancockii*), Epa (*Epipogium aphyllum*), Gan (*Gastrodia angusta*), Gel (*Gastrodia elata*), Gme (*Gastrodia menghaiensis*), Gpu (*Gastrodia pubilabiata*), Pmi (*Paphiopedilum micranthum*).

MTPTs varied widely among 14 orchid species, ranging from 0 in *G. menghaiensis* to 65 in *C. lancifolium* (**Figure 5A**, **Supplementary Table S9**). Analysis of MTPTs length revealed species-specific distribution patterns. Short fragments (0–500 bp) were most abundant in *Cymbidium* species and *D. hancockii*, whereas *P. micranthum* primarily contained 1000–2000 bp fragments. In *C. ensifolium* and *B. striata*, the fragments of 2000–5000 bp were predominant. MTPTs longer than 5000 bp were detected in *C. ensifolium*, *B. striata*, other *Cymbidium* species, and *G. pubilabiata*. The total MTPTs length also varied among species: *B. striata* had the greatest cumulative length, followed by *C. lancifolium*, whereas *G. angusta* had the shortest total length (excluding *G. menghaiensis*, which lacked MTPTs) (**Figure 5B**). The proportion of MTPTs in mitochondrial genomes ranged from 0 to 17.53%, with *C. ensifolium* exhibiting the highest fraction. No significant correlation was observed between total MTPTs length and mitochondrial genome size (r = −0.068, *p* = 0.82) (**Figure 5C**).

Functional assessment showed that some species contained 1–38 intact plastid genes within MTPTs, most frequently *psbF*, *atpE*, and *psbL* (**Figures 5D, E**). Photosystem-related genes were the most abundant (29.9%), followed by translation-related genes (29.3%) (**Figures 5D, E**). These results highlight the dynamic patterns of organelle DNA transfer and retention in orchid mitochondria.

### Selection pressure and nucleotide diversity in Orchidaceae organelle genomes

**Figure 6:**
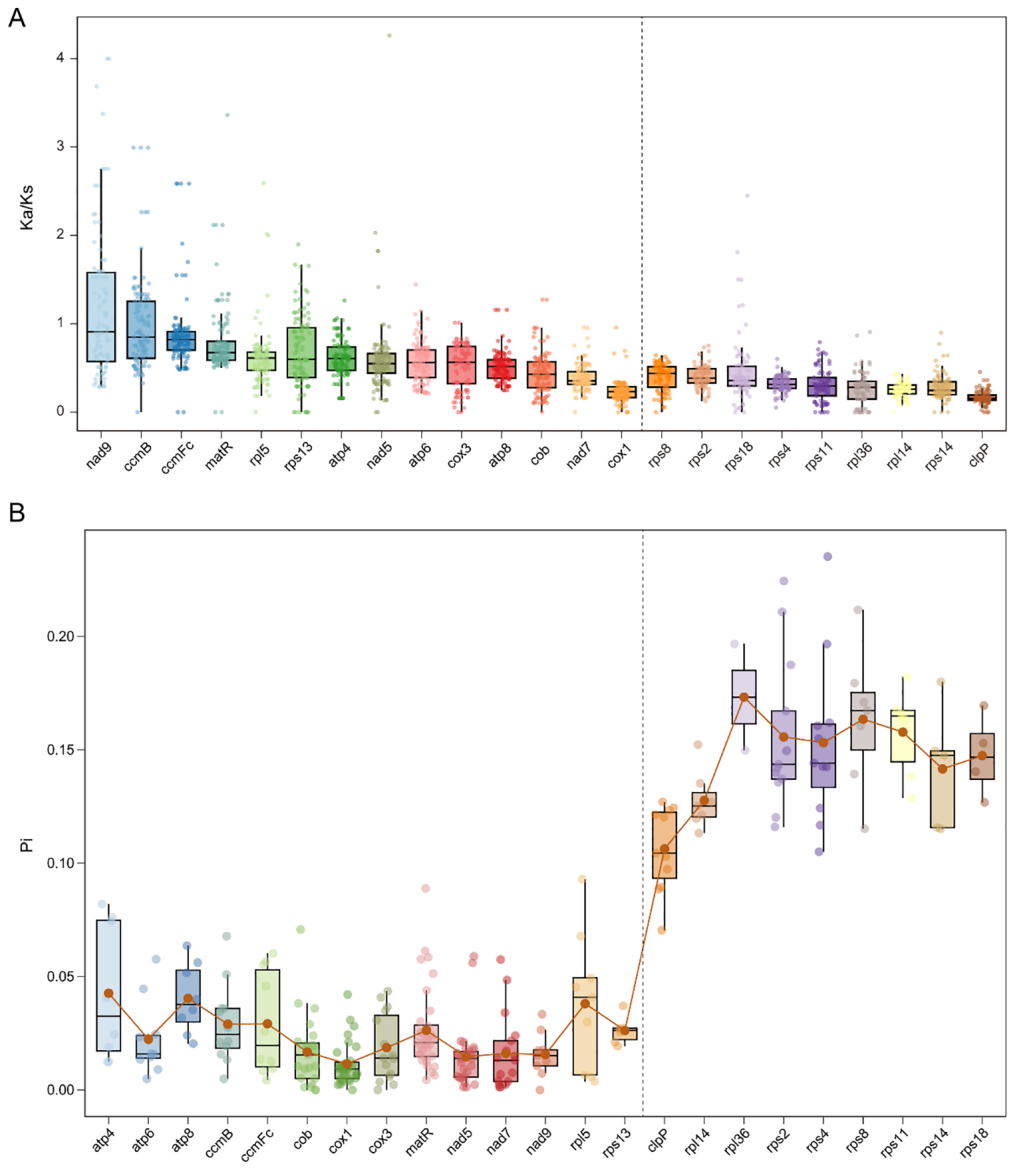
Evolutionary analysis of single-copy protein-coding genes shared between mitochondrial and plastid genomes of Orchidaceae. **(A)** Ka/Ks analysis of shared single-copy protein-coding genes. **(B)** Nucleotide diversity (Pi) analysis of the same genes. The dashed line separates mitochondrial genes (on the left) and plastid genes (on the right).

Selection pressure (Ka/Ks) and nucleotide diversity (Pi) were assessed for conserved organelle protein-coding genes. In mitochondrial genomes, 14 shared single-copy genes were examined (**Figure 6A**, **Supplementary Table S10**). Among these genes, *nad9* showed the highest Ka/Ks value, followed by *ccmB*, while *cox1* had the lowest. All genes exhibited Ka/Ks < 1, indicating strong purifying selection. Nucleotide diversity was similarly low, with *atp4* displaying the highest Pi value (0.0427), followed by *atp8* (0.0404), whereas *cox1* presented the lowest Pi value (0.0115) (**Figure 6B**, **Supplementary Table S11**). Additionally, all Pi values were below 0.05, indicating high sequence conservation in mitochondrial genomes.

In plastid genomes, nine shared single-copy genes were identified. Gene *rps8* displayed the highest Ka/Ks value, followed by *rps2*, whereas *clpP* had the lowest (**Figure 6A**). Although all genes exhibited Ka/Ks < 1, nucleotide diversity was markedly higher than in mitochondrial genes. The genes *rpl36* (0.1733) and *rps8* (0.1635) showed the greatest variability, while *clpP* had the lowest Pi value (0.1064) (**Figure 6B**). Overall, mitochondrial genes exhibit stronger purifying selection and greater sequence conservation, whereas plastid genes display substantially higher nucleotide diversity and faster evolutionary rates across Orchidaceae species.

### Phylogenetic relationships among Orchidaceae species

**Figure 7:**
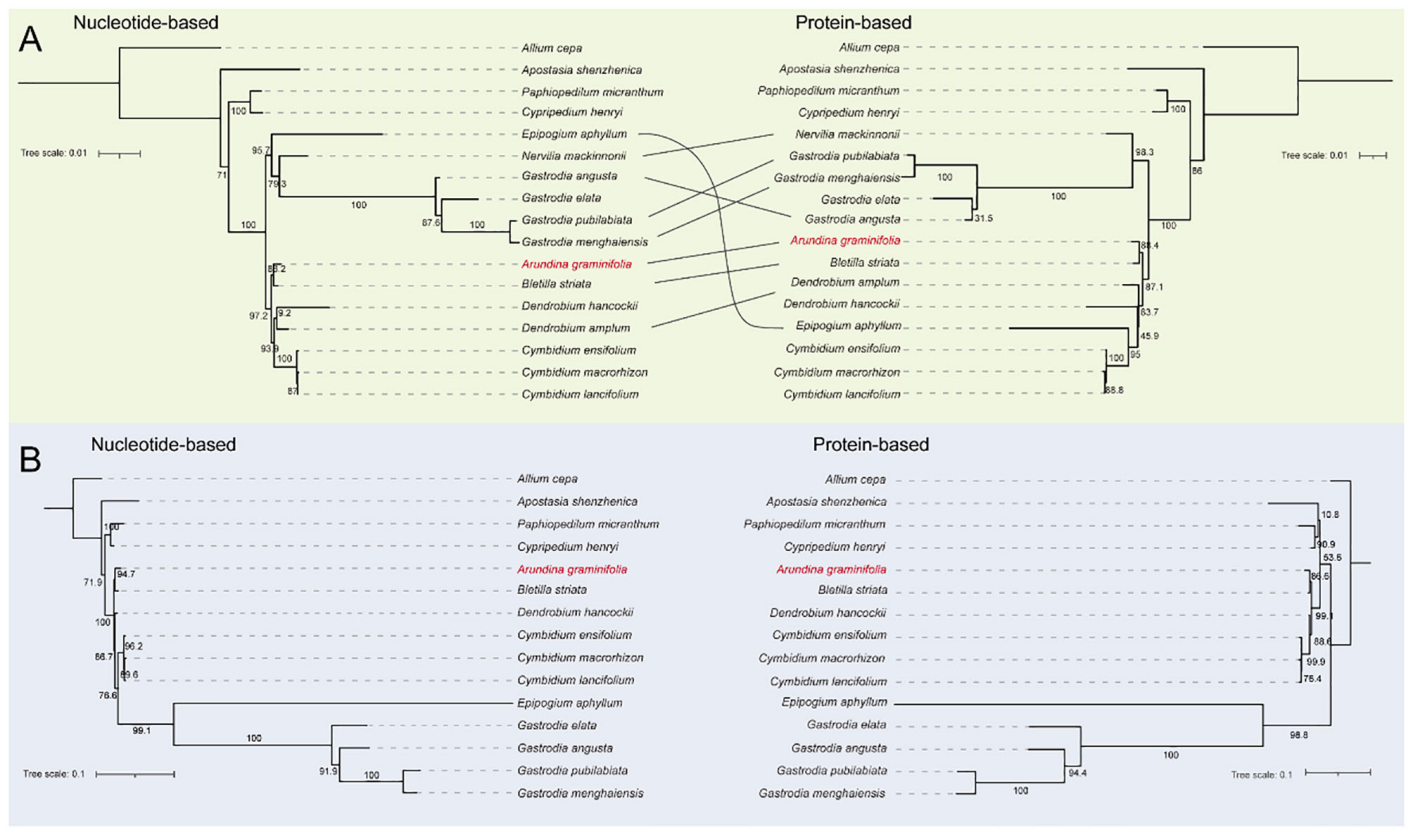
Phylogenetic analysis of Orchidaceae based on single-copy orthologs in mitochondrial and plastid genomes, with *Allium cepa* included as the outgroup. Maximum likelihood phylogenies of Orchidaceae species were inferred using nucleotide and protein sequences of single-copy orthologs in **(A)** mitochondrial genomes (17 species) and **(B)** plastid genomes (15 species).

Phylogenetic relationships among Orchidaceae species were inferred using shared single-copy genes from mitochondrial and plastid genomes. For mitochondrial genomes, 14 conserved single-copy genes from 17 species were analyzed at both nucleotide and protein levels (**Figure 7A**). The resulting trees showed highly consistent topologies with strong branch support. Basal lineages such as *Apostasia shenzhenica* and *P. micranthum* occupied stable positions, and *A. graminifolia* consistently clustered with *B. striata*, indicating a close evolutionary relationship.

Plastid phylogenies were constructed using nine shared single-copy genes from 15 species (**Figure 7B**). Nucleotide- and protein-based trees again produced congruent topologies, with most nodes receiving strong support. Major lineages occupied the same positions across trees, and *A. graminifolia* remained clustered with *B. striata*, consistent with the mitochondrial phylogeny. Overall, single-copy genes from both the mitochondrial and plastid genomes provide stable and reliable phylogenetic signals in Orchidaceae. The strong congruence between organelle-based phylogenies supports their effectiveness for resolving evolutionary relationships among orchid species.

### Comparative synteny of Orchidaceae mitogenomes

**Figure 8:**
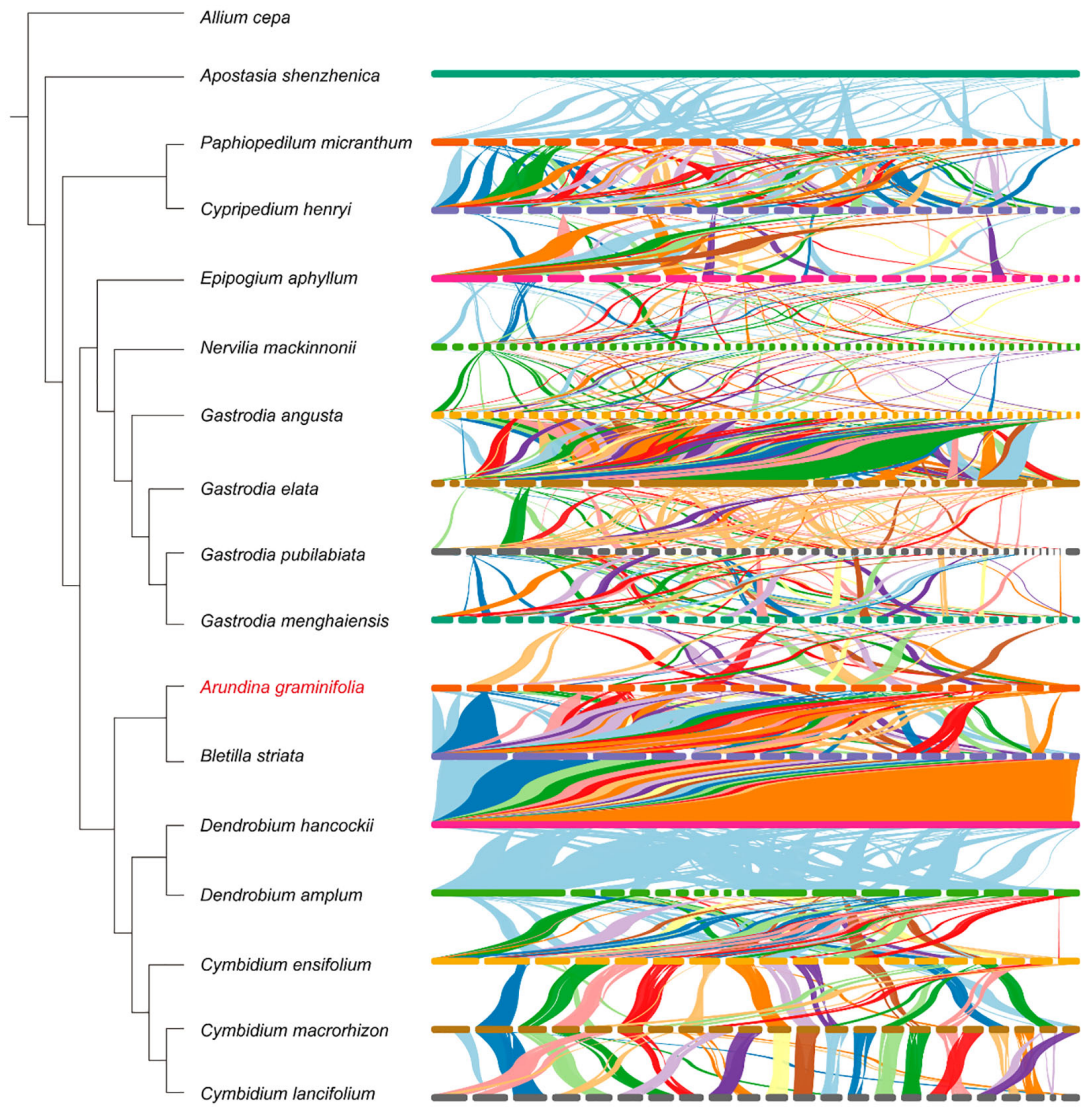
Synteny analysis of 16 Orchidaceae mitochondrial genomes. The synteny map highlights conserved and rearranged regions among the 16 orchid mitogenomes. Note that genomes and contigs are normalized to a uniform length for visualization purposes and are not to scale. Due to the integration of multiple species, some overlapping syntenic ribbons may appear clustered; for a detailed, deconstructed visualization of the homologous blocks between *B. striata* and *D. hancockii*, please refer to **Supplementary Figure S1**.

Structural conservation of mitochondrial genomes across Orchidaceae was assessed through synteny analysis of 16 species (**Figure 8**). Overall, mitochondrial genomes exhibited low synteny, with highly fragmented homologous blocks, indicating extensive genomic rearrangements across the family. Within the *Cymbidium* clade, synteny was comparatively higher than in other species, with several species sharing long, continuous homologous regions, suggesting a more conserved mitochondrial genome structure within this genus. However, noticeable block fragmentation and rearrangements were still presented, reflecting ongoing structural reorganization even among closely related species.

In contrast, mycoheterotrophic orchids such as *Gastrodia* species and *E. aphyllum* exhibited extremely low synteny with other taxa. Only a few short and discontinuous homologous blocks were detected, indicating that these lineages have undergone more extensive and complex mitochondrial genome restructuring. These patterns highlight substantial variation in mitochondrial genome architecture among Orchidaceae lineages, demonstrating that large-scale rearrangements have been pervasive throughout their evolutionary history.

## Discussion

### Robustness and reliability of organelle genome assemblies in *A. graminifolia*

The assembly of *A. graminifolia* organelle genomes using two independent *de novo* assemblers demonstrated overall high consistency, highlighting the robustness of the mitochondrial and plastid genome reconstructions. Minor differences were observed between the HiMT (Tang et al., 2025) and Tippo (Xian et al., 2025) assemblies, such as the presence of small linear fragments in Tippo and unaligned edges. This difference likely resulted from assembly artifacts rather than biologically meaningful variation, as these fragments lacked identifiable coding sequences and showed low sequencing support. Because both assemblers produced highly concordant and stable structural configurations, we did not further apply additional tools such as PMAT2 (Han et al., 2025) or Oatk (Zhou et al., 2025) for fine-scale assembly refinement. The strong agreement between HiMT and Tippo supports the reliability of the mitochondrial genome structure and gene content reported here. Furthermore, the identical plastid genome assemblies suggest a conserved plastome organization in *A. graminifolia*, reinforcing the suitability of the HiMT-based assembly as the reference for downstream comparative and evolutionary analyses.

### Contrasting evolutionary constraints of mitochondrial and plastid genomes

The mitochondrial genome of *A. graminifolia* exhibits a multichromosomal structure with extensive rearrangements, consistently across the selected Orchidaceae species (Yuan et al., 2018; Li et al., 2023; Yang et al., 2023; Shen et al., 2024; Soe et al., 2025; Wang et al., 2025). Highly fragmented homologous blocks indicate frequent recombination events and large-scale structural evolution. Notably, mycoheterotrophic lineages, including *Gastrodia* genus and *E. aphyllum*, display extremely low synteny with other taxa, suggesting lineage-specific expansions and complex restructuring of mitochondrial genomes. In contrast, plastid genomes of photosynthetic orchids tend to be conserved in overall architecture and gene content, although heterotrophic lineages often exhibit extensive genomic reduction (Raubeson and Jansen, 2005; Peng et al., 2022; Wen et al., 2022; Zhao et al., 2024). This stark difference reflects distinct evolutionary constraints that mitochondrial genomes are more prone to structural rearrangements, whereas plastid genomes are constrained by the functional requirements of photosynthesis and gene expression. The observed pattern aligns with previous findings in angiosperms, where plastid genomes evolve slowly in structure compared with recombination-active mitochondrial genomes (Sloan et al., 2018; Xu et al., 2021; Peng et al., 2022). Furthermore, the distribution of conserved versus variable genes supports the notion that functional essentiality contributes to evolutionary stability: genes involved in energy metabolism, such as ATP synthases and cytochrome c biogenesis genes, remain highly conserved in mitochondrial genome, whereas ribosomal protein genes show lineage-specific variation, reflecting relaxed constraints or potential functional redundancy (Gray et al., 1999; Maier et al., 2013; Skippington et al., 2017).

### Repetitive sequences and plastid DNA transfers as drivers of mitochondrial genome expansion

Repetitive elements play a crucial role in determining the size and structural complexity of the mitochondrial genome (Ouyang et al., 2025; Su et al., 2025). The analysis of SSRs and long repeats across 16 orchid species revealed substantial interspecific variation in number, length, and distribution, with the repeats of Class I being the most abundant. Species such as *G. pubilabiata* exhibit notable expansion of long repeats, which disproportionately contributes to genome size and likely facilitates homologous recombination and structural rearrangements. Additionally, MTPTs exhibit remarkable diversity among species, ranging from 0 to 65 fragments, with functional enrichment in genes related to photosynthesis and translation (Wang et al., 2012; Nhat Nam et al., 2024). The combined presence of repeats and MTPTs suggests a synergistic role in promoting genome expansion and rearrangement, which may provide a selective advantage by increasing genomic plasticity and buffering against deleterious mutations. The variation in repeat content and MTPTs abundance across species also indicates that mitochondrial genome evolution is influenced by both lineage-specific and ecological influences. For instance, mycoheterotrophic orchids typically contain fewer MTPTs but exhibit remarkable structural rearrangements, emphasizing the interplay between genome architecture and their life history traits (Zhao et al., 2024; Wang et al., 2025).

### Purifying selection, genetic diversity, and phylogenetic utility of single-copy genes

Selection analyses reveal that Orchidaceae mitochondrial single-copy genes are under strong purifying selection (Ka/Ks < 1) with low nucleotide diversity (Pi < 0.05), reflecting functional constraints and evolutionary stability. Conversely, Orchidaceae plastid single-copy genes, although under purifying selection, tend to show greater sequence variability compared with other regions, suggesting faster evolutionary rates in these loci (Jansen et al., 2007; Cole et al., 2018; Jiang et al., 2024). Phylogenetic reconstruction using single-copy genes generated congruent topologies for mitochondrial and plastid genomes, with *A. graminifolia* consistently clustering with *B. striata*, supporting their close evolutionary relationship (Li et al., 2019). Low synteny and fragmented homologous blocks in mitochondrial genomes, particularly in mycoheterotrophic orchids, underscore the dynamic nature of structural evolution (Lee et al., 2020; Zhao et al., 2024; Wang et al., 2025). Gene loss and duplication contribute to functional redundancy, potentially enhancing environmental adaptability (Costello et al., 2020; Li et al., 2024b). Collectively, these findings indicate that single-copy genes serve as reliable molecular markers for resolving Orchidaceae phylogeny (Li et al., 2019), whereas mitochondrial genome architecture reflects lineage-specific structural plasticity driven by both ecological adaptation and intrinsic genome dynamics (Wang et al., 2024a; Zheng et al., 2024).

## Conclusions

Orchidaceae organelle genomes exhibit contrasting evolutionary patterns. Mitochondrial genomes are structurally complex, multichromosomal, and under strong purifying selection, while plastid genomes are highly conserved yet genetically more diverse. Repetitive sequences and plastid DNA transfers drive mitochondrial genome expansion and rearrangement, whereas gene loss and duplication increase genomic plasticity. Single-copy genes provide robust markers for phylogenetic inference. These results provide a comprehensive understanding of the structural, functional, and evolutionary dynamics in Orchidaceae organelle genomes, laying a foundation for further studies on adaptation and diversification mechanisms.

## Data Availability

The data presented in the study are deposited in the NCBI GenBank repository, with the mitochondrial genome of *A. graminifolia* available as multiple circular molecules under accession numbers PX694305–PX694324, and the plastid genome under accession number PX694325.
