# Supplementary material for: Comparative organellar genomics of Arundina graminifolia: mitochondrial complexity and plastid conservation in Orchidaceae
Source: Front Plant Sci. 2026 Feb 12;17:1756243. doi: 10.3389/fpls.2026.1756243 (PMC12936008; doi:10.3389/fpls.2026.1756243)
Supplement: Supplementary file 1 [file Table1.docx]

**
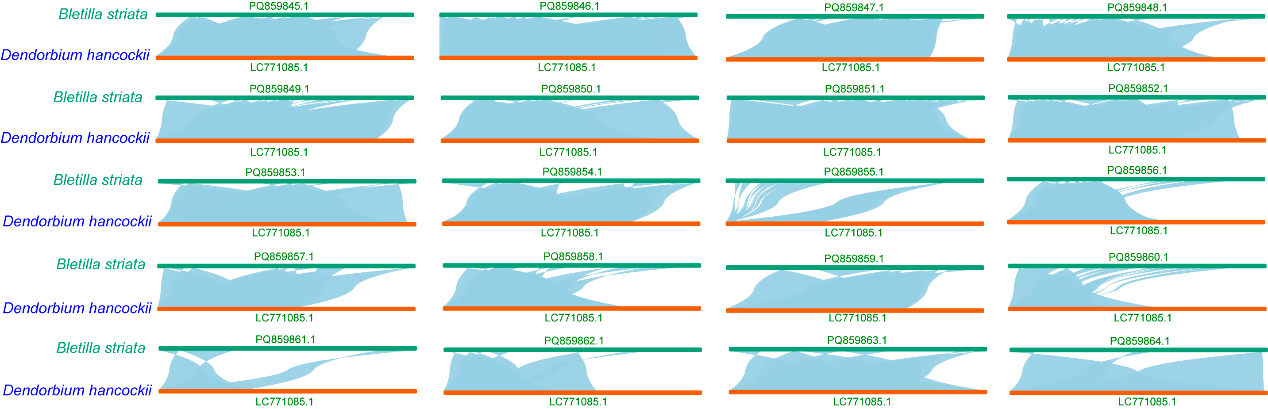
Supplemental Figure 1. Visualization of homologous blocks between *Bletilla striata* and *Dendrobium hancockii* mitogenomes.** To address the visualization constraints in Figure 8, where overlapping layers obscured individual alignments, each contig of the *B. striata* mitogenome was visualized separately against the *D. hancockii* mitogenome. This deconstructed view clearly displays the extensive homologous regions between the two species, confirming that the synteny patterns observed in the integrated map are based on valid sequence homology rather than computational artifacts. Note that genomes and contigs are normalized to a uniform length for visualization purposes and are not to scale.
